# Cardiac Myxoma Presenting as Dyspnea after Cesarean Delivery

**DOI:** 10.1155/2012/487385

**Published:** 2012-06-14

**Authors:** Allison Wyman, William Hurd, Justin Lappen

**Affiliations:** ^1^Department of Obstetrics and Gynecology, University Hospitals Case Medical Center, 11100 Euclid Avenue, Cleveland, OH 44106, USA; ^2^School of Medicine, Case Western Reserve University, Cleveland, OH 44106, USA

## Abstract

*Introduction.* Dyspnea during pregnancy and in the immediate postpartum or postoperative period is a relatively common symptom that can be an early sign of a life threatening condition. The differential diagnosis is broad and can represent a wide variety of underlying etiologies. Cardiac tumors are one of the rarest causes of dyspnea in a reproductive age women during the postpartum period. *Case Presentation.* 42-years old G_7_P_1051_ presented with acute dyspnea postoperatively after an elected uncomplicated repeat cesarean section and tubal ligation. The patient was diagnosed with a large left atrial cardiac myxoma and required urgent cardiothoracic surgery. *Conclusion.* The following case illustrates how a standard response to a common postpartum symptom, dyspnea, can divert and distract from less common exam findings. A careful, stepwise evaluation of symptoms and related findings will usually determine the underlying cause so that appropriate and timely treatment can be initiated.

## 1. Introduction

Acute dyspnea after delivery is a common symptom that can signify a wide variety of underlying etiologies. Cardiac and pulmonary causes, such as thromboembolism, pulmonary edema, or cardiomyopathy, are perhaps the more common life-threatening conditions. However, uncommon causes must also be considered in the differential of patients with dyspnea following delivery.

Cardiac tumors are extremely rare in reproductive age women, with an overall incidence of less than 0.2% [1]. Left atrial myxoma is the most common cardiac tumor representing 70% of all benign heart neoplasms [1, 2]. Diagnosing any cardiac tumor in a symptomatic postpartum patient can be challenging because of their infrequent occurrence, unpredictable clinical presentation, and symptoms that mimic the more common postpartum morbidities. The following case illustrates how a standard response to a common postpartum symptom, dyspnea, may divert and distract from less common exam findings such as a diastolic murmur and a soft tissue mass on CT scan. 

## 2. Case Presentation

A 42-year-old G_7_P_1051_ presented at 39 weeks estimated gestational age for a scheduled elective repeat low transverse cesarean section with bilateral tubal ligation. Two years earlier, she had undergone a low transverse cesarean section for arrest of descent. Following that delivery, she had an episode of moderate dyspnea that spontaneously resolved without evaluation or treatment. Her medical and surgical history was otherwise unremarkable. During the current pregnancy, her antenatal course was complicated by class A1 gestational diabetes mellitus treated with diet. 

On the day of her cesarean delivery, her vital signs and physical exam were normal, and her hemoglobin was 12.6 gm/dl. Her surgical procedures were unremarkable and the estimated blood loss was 600 mL. 

Approximately twelve hours after delivery, the patient experienced the sudden onset of dyspnea. Initially, she had no other symptoms such as chest pain, cough, palpitations, or calf pain. On physical examination, she had moderate respiratory distress with tachypnea (RR 24/min) and tachycardia (HR 107 bpm). The patient's pulmonary exam revealed tachypnea with use of accessory muscles, shallow breaths, and coarse crackles bilaterally at the bases. The cardiovascular exam demonstrated tachycardia with regular rhythm and a diastolic murmur was appreciated. No S3 or S4 heart sounds were noted at the time. The patient required oxygen supplementation via nasal cannula at 2 liters per minute to maintain saturation at > than 95%. Laboratory work was sent and her hemoglobin was reassuring at 12.0 gm/dl.

 Several hours later, the patient continued to experience dyspnea with an increased demand for supplemental oxygen, requiring a high flow face mask at 10 liters per minute. Vital sign abnormalities progressed to a more concerning state of respiratory distress as her heart rate increased to over 115 bpm and her respiratory rate to > 26/min. A diagnosis of pulmonary embolism (PE) was considered and a spiral CT was ordered. The preliminary read of the CT scan demonstrated small bibasilar pleural effusions with extensive parenchymal airspace disease and multifocal areas of patchy, bilateral consolidation with relative sparing of the periphery in the upper lobes, consistent with underlying infection, hemorrhage, evolving ARDS, or progressive pulmonary edema. No evidence of a PE was noted on the preliminary reading. 

Over the next three hours, the severity of her tachycardia and tachypnea increased (HR > 120 bpm and RR > 28/min). She developed orthopnea and hemoptysis suggestive of heart failure. Administration of intravenous furosemide resulted in partial resolution of her symptoms. Cardiac echocardiogram was obtained for a tentative diagnosis of postpartum cardiomyopathy. However, rather than an enlarged heart, the echocardiogram revealed a large left atrial mass obstructing the mitral valve and resulting in functional mitral stenosis.

The patient underwent an emergent thoracotomy approximately 48 hours after her cesarean delivery. A 5 cm, intracardiac tumor consistent with an atrial myxoma was found adherent to the inferolateral left atrium and the annulus of the mitral valve. The tumor was completely resected and sent for pathological examination. Microscopic analysis revealed a benign atrial myxoma.

The patient was transferred to SICU postoperatively in stable condition and was monitored closely. Over the next two days, despite the patient being initially extubated and clinically stable, the patient's condition deteriorated and she developed signs of cardiogenic shock. A transesophageal echocardiogram revealed evidence of severe aortic insufficiency and the patient was emergently taken back to the operating room 48 hours after her initial cardiac surgery. 

At the time of her second open heart surgery, she was found to have disruption of the noncoronary posterior cusp of the aortic valve which had separated from its base at the aortic root likely secondary to aggressive resection of the myxoma during the initial surgery resulting in severe aortic insufficiency. The patient underwent aortic root replacement with the insertion of a bioprosthetic aortic valve. 

After surgery, the patient required initial hemodynamic support with an intra-aortic balloon pump and pressors. After a slow recovery, she was discharged to home twenty days following her second cardiac surgery. The patient did well at home requiring 20 mg of furosemide and 81 mg of aspirin daily as her only medications. At two months, a follow-up echocardiogram showed no signs of heart failure or aortic insufficiency with an estimated ejection fraction measuring 60%–65%. The patient has resumed all physical activity at the level prior to her cardiac procedures and has no long term sequelae. 

## 3. Discussion 

Dyspnea during pregnancy and in the immediate postpartum or postoperative period is a relatively common symptom that can be an early sign of a life-threatening condition. The differential diagnosis is broad, and should include iatrogenic fluid overload, pulmonary embolism, pneumonia, flash pulmonary edema from severe preeclampsia, and amniotic fluid embolism. Less common causes include congestive heart failure related to prior cardiac disease, postpartum cardiomyopathy, or exacerbations of previously unrecognized cardiopulmonary disease.

The differential diagnosis for dyspnea following a cesarean section for this case included fluid overload, pneumonia, a pulmonary embolism, pulmonary edema from severe preeclampsia, and peripartum cardiomyopathy. Iatrogenic fluid overload can occur after any operative procedure, but the dramatic change in fluid balance in the immediate postpartum period can complicate a routine clinical picture. This condition is high on the differential, but often diagnoses with graver consequences should be ruled out prior to giving furosemide and waiting for a clinical response. 

Pulmonary embolism can be life threatening. Timely diagnosis with initiation of the proper therapy is crucial for an optimal outcome. Pregnancy is a risk factor for a thrombotic event and the incidence of a pulmonary embolism is higher postpartum than in the antepartum period. Classically, the clinical signs of a pulmonary embolism are the acute onset of tachycardia and tachypnea with increased oxygen requirements. The presence of both these symptoms in our patient prompted her initial workup for a PE via spiral CT scan. 

Dyspnea from pneumonia is also common and presents with an elevated temperature, fatigue, rigors, and leukocytosis, none of which were found in this patient. Pulmonary edema secondary to severe pre-eclampsia is another cause of dyspnea during pregnancy or immediately postpartum. However, our patient had neither elevated blood pressures nor proteinuria. Amniotic fluid embolism should be considered with the sudden onset of dyspnea in the postpartum period. Fortunately, our patient did not have this anaphylaxis-related condition which manifests as otherwise unexplained cardiorespiratory collapse and is confirmed at autopsy by the presence of fetal squamous cells in the maternal pulmonary circulation. 

The appropriate evaluation for a woman with postpartum dyspnea depends on the severity and the presence of other symptoms. A combination of tachypnea, tachycardia, and decreased oxygen saturation made us suspect PE, which we excluded with a spiral CT scan. In the absence of a PE, her diastolic cardiac murmur and bi-basilar pulmonary crackles suggested a cardiac etiology, and thus a cardiac echocardiogram was obtained and revealed her intracardiac mass. 

Cardiac tumors are one of the rarest causes of postpartum dyspnea. Cardiac myxomas have only been reported during pregnancy in 17 previous cases [2, 3]. The majority of the published cases are sporadic with the left atrium being the most common location. Left atrial myxomas are more frequently seen in women than in men and the mean age of onset for women is between 30 and 60 years, as seen with our patient [2]. However, 7%–10% of myxomas worldwide are familial, and patients with familial myxomas may present with a constellation of extracardiac symptoms [4]. 

First described in 1973, syndromes with cardiac myxomas have been referred to by a number of names within the literature including; syndrome myxoma, Swiss syndrome, NAME (nevi, atrial myxoma, myxoid neurofibroma, ephelides), LAMB (lentigines, atrial myxoma, mucocutaneous myxoma, blue nevi), and Carney syndrome [5]. Although up to 90% of cardiac myxomas present after 40 years of age, familial cardiac myxomas can present as early within teenage years [4]. Carney's complex syndrome is thought to be an autosomal dominant inheritance and is the most studied of all the familial syndromes. Carney's complex syndrome is characterized by the association of extra cardiac findings including cutaneous pigmentation, fibromyxoid tumors of the skin, and endocrine over activity [6]. New studies have identified genetic mutations that are promising for future genetic testing [6]. Our patient did not possess any extra-cardiac findings on physical exam to suggest a familial syndrome. However, if there is any evidence of extra-cardiac manifestations, it is imperative to evaluate for genetic disorders. 

Cardiac myxomas produce symptoms by several mechanisms, but usually only after they have become relatively large enough in size to cause obstruction, which is approximately 70 grams in weight [7]. The most common clinical symptoms from a myxoma include dyspnea, dizziness, or syncope reflecting a cardiac outflow obstruction with subsequent congestive heart failure [8]. Less commonly, symptoms arise from arterial or venous emboli with resulting cerebral ischemia or pulmonary embolism [7, 9].

Postpartum dyspnea is common and has a wide variety of possible etiologies. A careful, stepwise evaluation of symptoms and related findings will usually determine the underlying cause so that appropriate and timely treatment can be initiated.

## Supplementary Material

Low attenuation structure adjacent to the interatrial septum of the right atrium consistent with a cardiac myxoma measuring approximately 3.3 x 2.0 cm in size was described as an addendum to the final read.
