# Supplementary material for: Cardiac Myxoma Presenting as Dyspnea after Cesarean Delivery
Source: Case Rep Med. 2012 Jun 14;2012:487385. doi: 10.1155/2012/487385 (PMC3384962; doi:10.1155/2012/487385)
Supplement: Supplementary file 1 — Low attenuation structure adjacent to the interatrial septum of the right atrium consistent with a cardiac myxoma measuring approximately 3.3 x 2.0 cm in size was described as an addendum to the final read. [file 487385.f1.doc]

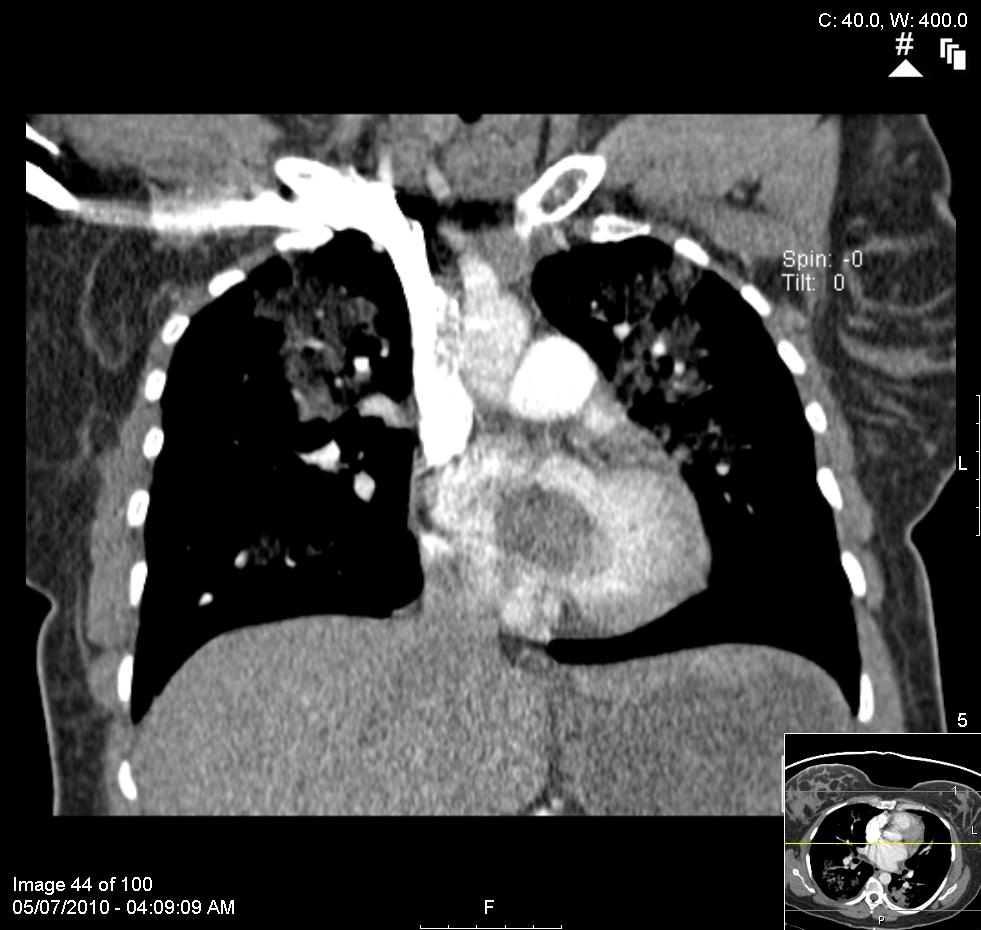


**Figure 1:** Low attenuation structure adjacent to the interatrial septum of the right atrium consistent with a cardiac myxoma measuring approximately 3.3 x 2.0cm in size was described as an addendum to the final read.
